# Amino acid substitutions in the neuraminidase protein of an H9N2 avian influenza virus affect its airborne transmission in chickens

**DOI:** 10.1186/s13567-014-0142-3

**Published:** 2015-04-18

**Authors:** Jing Lv, Liangmeng Wei, Yan Yang, Bingxiao Wang, Wei Liang, Yuwei Gao, Xianzhu Xia, Lili Gao, Yumei Cai, Peiqiang Hou, Huili Yang, Airong Wang, Rong Huang, Jing Gao, Tongjie Chai

**Affiliations:** College of Animal Science and Veterinary Medicine, Shandong Agricultural University; Sino-German Cooperative Research Centre for Zoonosis of Animal Origin Shandong Province; Key Laboratory of Animal Biotechnology and Disease Control and Prevention of Shandong Province, Shandong Agricultural University, Daizong Street 61, Taian, 271018 China; Taian Municipal Center for Disease Control and Prevention, Changcheng Street, Taian, 271000 China; College of Life Science, Shandong Agricultural University, Taian, 271018 China; Qingdao Chia Tai Co., Ltd, Qingwei Street, Qingdao, 266109 China; Institute of Military Veterinary PLA, Qinglong Street 1068, Changchun, 130000 China; School of dentistry and oral health Taishan Medical University, Tai’an, 271000 China

## Abstract

Cases of H9N2 avian influenza virus (AIV) in poultry are increasing throughout many Eurasian countries, and co-infections with other pathogens have resulted in high morbidity and mortality in poultry. Few studies have investigated the genetic factors of virus airborne transmission which determine the scope of this epidemic. In this study, we used specific-pathogen-free chickens housed in isolators to investigate the airborne transmissibility of five recombinant H9N2 AIV rescued by reverse genetic technology. The results show that airborne transmission of A/Chicken/Shandong/01/2008 (SD01) virus was related to the neuraminidase (NA) gene, and four amino acid mutations (D368E, S370L, E313K and G381D) within the head region of the SD01 NA, reduced virus replication in the respiratory tract of chickens, reduced virus NA activity, and resulted in a loss of airborne transmission ability in chickens. Similarly, reverse mutations of these four amino acids in the NA protein of r01/NASS virus, conferred an airborne transmission ability to the recombinant virus. We conclude that these four NA residues may be significant genetic markers for evaluating potential disease outbreak of H9N2 AIV, and propose that immediate attention should be paid to the airborne transmission of this virus.

**Electronic supplementary material:**

The online version of this article (doi:10.1186/s13567-014-0142-3) contains supplementary material, which is available to authorized users.

## Introduction

Cases of poultry infected with H9N2 avian influenza virus (AIV) have occurred repeatedly in many countries since its isolation in America in 1966 [1-4]. H9N2 AIV mainly infected feral birds and wild ducks in South America, and were unable to form a stable lineage in poultry [5,6]. H9N2 AIV infection of chickens, turkeys, pheasants, and domestic ducks has been reported throughout Asia, the Middle East, Europe and Africa from 1995 to 1997 [5,7-9]. H9N2 AIV isolated from diseased chickens in Guangdong province in 1994, represented the first documented case of H9N2 AIV in mainland China, and since 1998 the virus has spread widely [10-13]. Although it was considered a low pathogenic AIV, H9N2 caused high morbidity and mortality in poultry when co-infected with other pathogens [3,14]. More importantly, it was reported that H9N2 AIV had crossed the species barrier, infecting humans in Hong Kong and Guangzhou, China [15,16].

The segmented RNA genome of AIV allows genetic reassortment among different subtypes. The major human influenza pandemics have resulted in high mortalities, and genetic analysis of the infecting strains have indicated that the genes are partially derived from AIV [17-21]. These studies highlight the need for greater research into virus gene mutations and recombinations, and their subsequent effects on virus transmission.

H9N2 AIV transmission among chickens occurs by direct contact and aerosols, with the latter responsible for its prevalence beyond 1998 [22,23]. The NA gene of influenza virus has been shown to play an important role in virus transmission, influencing viral enzyme activity and transmissibility [23,24]. Thus, further study of the NA gene is required to help elucidate the mechanism of airborne transmission of H9N2 AIV in poultry.

Our laboratory established an experimental animal model using three groups of specific-pathogen-free (SPF) chickens housed in isolators. The groups were designated inoculated, direct contact and aerosol contact, and were used to study the generation, spread and infection progression of AIV by aerosols. The results showed that the A/Chicken/Shandong/01/2008 (SD01) virus could be transmitted among chickens via aerosols [22]. A/Chicken/Guangdong/SS/94 (SS94) AIV, the earliest H9N2 virus isolated from diseased chickens in mainland China, is not transmitted by aerosol among chickens, while A/Chicken/Shanghai/F/98 (F98) could be transmitted [23]. Interestingly, substituting the NA gene of F98 with that of SS94 completely abolished the airborne transmission of the recombinant virus [23]. There are four identical amino acids in the NA head region of SD01 and F98 viruses, but they were different from the SS94 virus. These differences are at residues 313, 331, 368 & 370 and 381 (N2 number). Amino acids 368 & 370 belong to the 366–373 peptide loop which was one of hemadsorbing (HB) sites affecting neuraminidase activity [25]. However, amino acids 313–381 are on the opposite side of the globular head. To determine whether NA also influences SD01 transmission in chickens, we designed a series of mutations in the NA gene using reverse genetics systems, and evaluated their effects on airborne transmission.

## Materials and methods

### Virus and cell culture

H9N2 AIV A/Chicken/Shandong/01/2008 (SD01) H9N2 AIV was isolated from diseased chickens as described previously [22]. Viruses were propagated in 9-day-old SPF embryonated chicken eggs and stored at −80 °C. The Reed-Muench method [26] was used to calculate the 50% egg infective doses (EID_50_).

Madin Darby canine kidney (MDCK) and 293 T cells were maintained in Dulbecco’s minimum essential medium (Gibco-BRL, Grand Island, NY, USA) supplemented with 10% fetal bovine serum, penicillin (100 units/mL), and streptomycin (100 mg/mL), and incubated at 37 °C with 5% CO_2_.

### Site-directed mutagenesis and virus generation

The QuickChange II site-directed mutagenesis kit (Stratagene, La Jolla, CA, USA) was used to create site-specific mutations in the NA genes of H9N2 AIV. The primers used for mutagenesis are listed in Table 1. The eight-plasmid system was used to generate H9N2 reassortant viruses according to published methods [27,28]. Recombinant plasmids were purified using the Genopure Plasmid Midi Kit (Roche, Basel, Switzerland). Recombinant rSD01, r01/NASS, r01/NAHB, r01/NA381 and r01/NASS-381 viruses were rescued according to previously reported protocols with minor modifications [29,30]. Briefly, eight plasmids (0.5 μg each) of influenza virus were transfected into 5:1 mixed 293 T and MDCK cells using Lipofectamine^TM^ 2000 Transfection Reagent (Invitrogen, Carlsbad, CA, USA). OPTI-MEM I (Gibco, USA) was used to substitute the transfection mixture after incubation for 6 h at 37 °C. Twelve hours later, OPTI-MEM I containing 0.5 μg/mL TPCK-trypsin and antibiotics was added. This medium was collected at 48 h post transfection and propagated in 9-day-old embryonated SPF chicken eggs. Rescued viruses were verified by sequencing the complete genes to ensure the induced mutations and the absence of unwanted mutations. The rescued viruses were titrated and stored as described above.

**Table 1:** **Primers used to generate mutations in the NA gene of SD01 virus**

**Mutation**	**Primer sequence (5’-3’)**	
	**Forward**	**Reverse**
D368E&S370L	TGGATGGGACGGACAATCAAAGA**G**GATT**T**ACGCTCAGGTTA	TAACCTGAGCGT**A**AATC**C**TCTTTGATTGTCCGTCCCATCCA
E313K	GTTCTATATATAAATATGGCAGATTATAGTATT**A**AGTCCAGTTATGTGTGC	GCACACATAACTGGACT**T**AATACTATAATCTGCCATATTTATATAGAAC
G381D	CTTTCAGGGTCGTTG**A**TGGTTGGACCACGGC	GCCGTGGTCCAACCA**T**CAACGACCCTGAAAG

The changed nucleotides are in boldface.

### Infection and transmission in chickens

Studies using H9N2 low pathogenic AIV were conducted in a Biosecurity Level 2+ laboratory approved by the China National Accreditation Service for Conformity Assessment. All animal studies were carried out in strict accordance to the guidelines of Laboratory Animal Management by the National Council for Science and Technology.

For virus transmission studies, ten 4-week-old white leghorn SPF chickens (Chinese Academy of Agricultural Sciences) were inoculated intranasally with 10^7^ EID_50_ of infectious allantoic fluid and placed in isolator A. A further ten animals, representing direct contact, were introduced into the same isolator 24 h later. Ten animals (aerosol contact) were placed in different cages respectively in isolator B. Isolators A and B were joined by a tube (length: 1 m; diameter: 0.08 m) to allow air flow from A to B. Oropharyngeal and cloacal swab samples, collected at 2-day intervals for all chickens, were immersed in 1 mL sterile PBS. The solutions were filtered through 0.22 μm Millex syringe filters (Millipore Corp., Bedford, MA, USA) and cultivated in 9-day-old embryonated SPF chicken eggs. Sera samples were collected at 7, 14 and 21 days post inoculation (dpi) and seroconversion confirmed by HI assay according to the OIE protocol.

Air samples were collected simultaneously at 2-day intervals from the space of two isolators as previously described [31]. The SPF isolators (Tianjin Jinhang Pure Air Condition Engineering Co., Tianjin, China) were 222 cm (length) × 86 cm (width) × 103 cm (height), and the airflow was 0.1 m/s. The environmental conditions for this study were 20–22 °C and 45–50% relative humidity.

### Tropism for the respiratory tract of chickens

The replication ability and tissue tropism of H9N2 AIV, was investigated in five 4-week-old white leghorn SPF chickens inoculated intranasally with 10^7^ EID_50_ virus in PBS (0.1 M, pH 7.2). Three chickens were euthanized at 5 dpi and the trachea and lungs were collected for virus titration in SPF embryonated chicken eggs from initial dilutions of 1:10 as previously described [32]. Morbidity and mortality of all chickens were monitored for 2 weeks for signs of disease and death, and their sera were collected at 14 dpi and were evaluated for seroconversion by an HI assay.

### NA activity assays

Virus copy number used for the dose of comparable virus in NA enzymatic assays was calculated by real-time RT-PCR [31]. Virus was incubated with an equal volume (50 μL) of 0–400 μM 4-methylumbelliferyl N-acetylneuraminic acid (4-MUNANA; Sigma-Aldrich, St.Louis, MO, USA) at 37 °C in U-bottomed microtiter plates [33,34]. The fluorescence of liberated 4-methylumbelliferone was measured every 68 s for 45 min on a Spectra MaxM2/Me2 microplate detection system (Molecular Devices, LLC, Sunnyvale, California, USA) with excitation and emission wavelengths of 355 and 460 nm, respectively. Michaelis-Menten constant (*K*m) and maximum velocity of substrate conversion (*V*max) were calculated using the Michaelis-Menten nonlinear regression model (Prism; GraphPad, San Diego, CA, USA).

HA assays were performed on serial two-fold dilutions of viruses in PBS to determine the length of virus elution from agglutinated chicken red blood cells (CRBC) [34]. Microtiter plates with serial 50 μL mixtures were held at 4 °C for 1 h for virus adsorption to CRBC. The plates were transferred to a 37 °C water bath for virus elution. Samples were monitored for a minimum of 48 h, once a decrease in HA titer, coincident with NA-mediated virus elution, was observed.

## Results

### NA is important for aerosol transmission of H9N2 AIV in chickens

**Figure 1:**
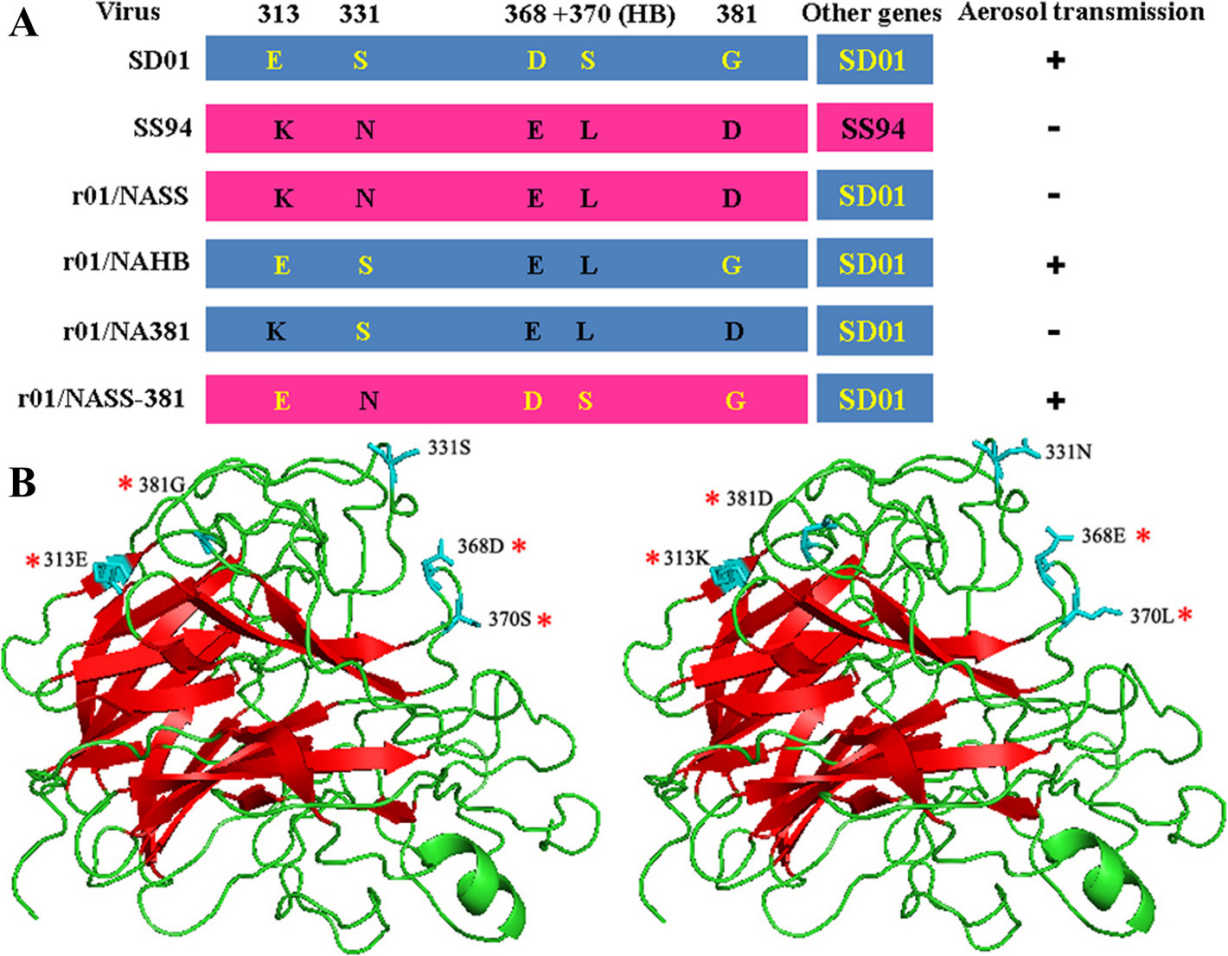
**Gene schematic diagrams and 3D-structure of H9N2 neuraminidase used in this study. (A)** Gene schematic diagrams of SD01, SS94 and recombinant virus. The blue and red bars indicate the genes originating from SD01 and SS94, respectively. The positions of four amino acid regions in the NA gene are shown at the top of the diagram and differences in the mutants are shown as amino acid abbreviation. Amino acids in NA of SD01 and SS94 are marked up by yellow and black letters respectively. + indicates that the virus could be transmitted among chickens via aerosols, − indicates could not. **(B)** The 3D-structure of H9N2 neuraminidase generated using PyMOL software shows the locations of mutations (PDB access number: 1ivd). The left is the structure before the mutation, and the right is the structure after the mutation. * indicates the positions that will be studied. Amino-acids 368–370 are close to the HB site, while 313–381 are on the opposite side of the globular head.

To determine whether NA also influences SD01 transmission in chickens, we first tested two recombinant viruses: recombinant SD01 (rSD01) virus recovered using reverse genetics; and r01/NASS virus, in which the NA of virus SD01 was replaced by that of SS94 (Figure 1A).

**Figure 2:**
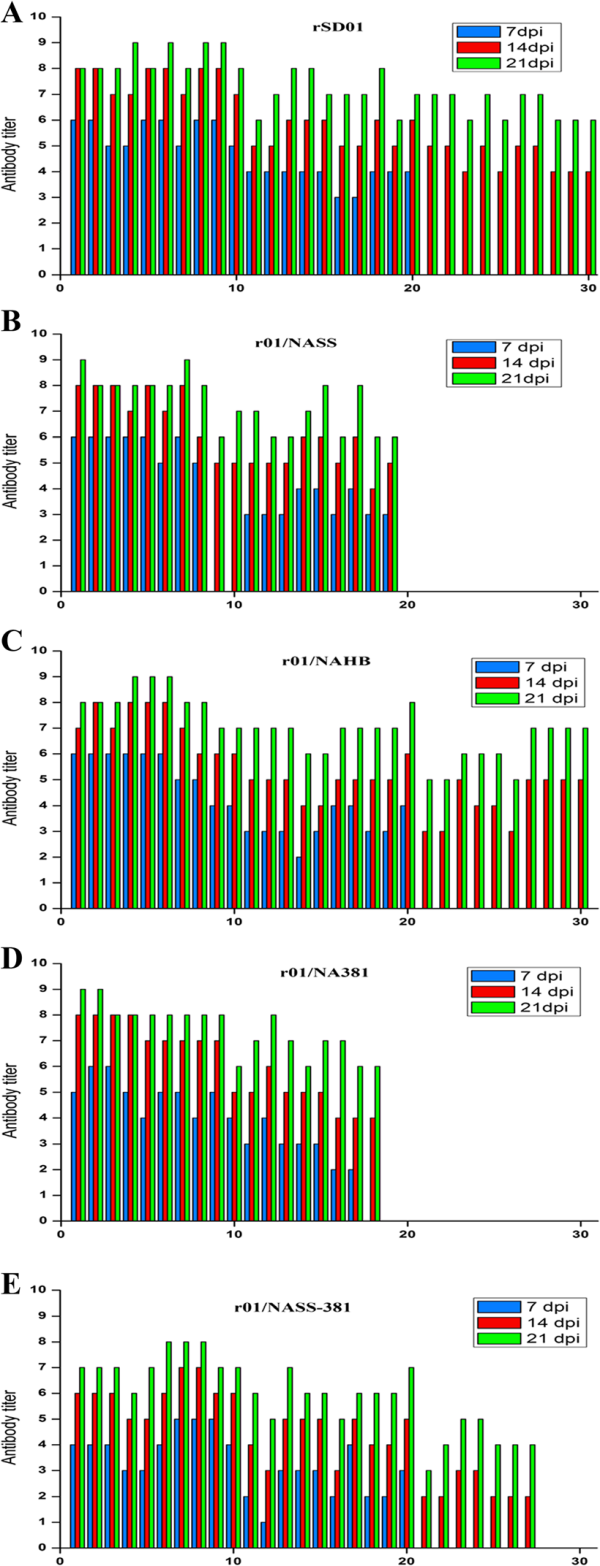
**Seroconversion of chickens in the transmission experiments.** In every independent experiment, sera of ten chickens in every group were collected at 7 day intervals and seroconversion was confirmed by hemagglutination inhibition (HI) assay. Each color bar represents the antibody titers of every chicken. Repeat seroconversion experiments of r01/NASS and r01/NA381 were performed. The results also indicate that no seroconversion was observed in aerosol contact chickens. 1–10 represents ten inoculated chickens, 11–20 represents ten direct-contact chickens and 21–30 represents ten aerosol-contact chickens. The blue, red and green colors express antibody titers detected on 7, 14 and 21 dpi respectively.

**Figure 3:**
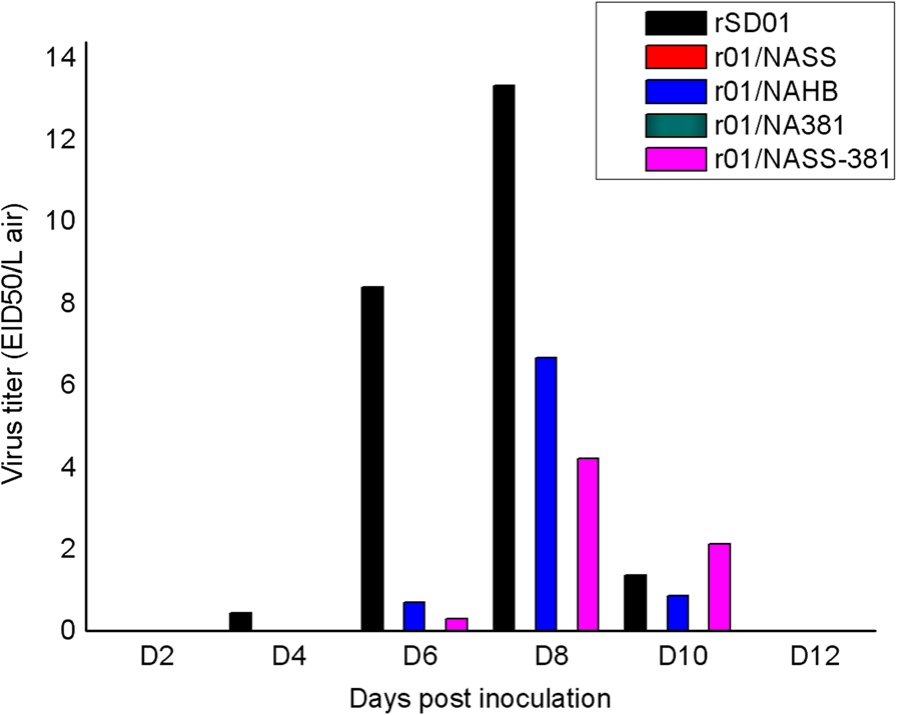
**Virus titers of airborne H9N2 AIV in an isolator.** Air samples were collected simultaneously from the space of two isolators using an AGI-30 liquid sampler operated continuously for an optimized time of 30 min at an airflow rate of 12.5 L/min. Virus titer in the air was expressed as values of EID_50_/L air. Each color bar represented the every recombinant virus concentration in the air collected every two days from the beginning of 2 dpi in an independent experiment. No airborne virus was detected in the experiment for r01/NASS and r01/NA381 viruses.

As shown in Table 2, virus was detected in the oropharyngeal and cloacal swab samples of all rSD01 and r01/NASS inoculated chickens at 4 dpi. rSD01 and r01/NASS virus were also detected in direct contact chickens at 2–14 dpi or 4–10 dpi. rSD01 virus was detected in the oropharyngeal and cloacal swab samples of six aerosol contact chickens at 4 dpi, and ten chickens at 8 dpi. No virus was detected in aerosol contact chickens for r01/NASS. Seroconversion was observed for all inoculated chickens, with average antibody titers increasing until 21 dpi (Figures 2A and B). Seroconversion was also observed for rSD01 infected direct contact chickens and for aerosol contact chickens (Figure 2A). In contrast, seroconversion was not observed for aerosol contact chickens exposed to r01/NASS (Figure 2B). Virus aerosols were detected in the air of rSD01 isolators from 4–10 dpi, but not for r01/NASS isolators (Figure 3). These results indicate that recombinant virus rSD01 was detected in the air and transmitted by aerosols between chickens, but recombinant virus r01/NASS, in which the NA of virus SD01 was replaced by that of virus SS94, was not detected in the air and was not aerially transmitted. These results suggest that the NA gene is an important determinant in virus transmission by aerosols.

**Table 2:** Number of chickens infected by recombinant virus in independent experiments*

**Dpi**	**rSD01**			**r01/NASS**			**r01/NAHB**			**r01/NA381**			**r01/NASS-381**		
	**Inoculated**	**Direct-contact**	**Aerosol-contact**	**Inoculated**	**Direct-contact**	**Aerosol-contact**	**Inoculated**	**Direct-contact**	**Aerosol-contact**	**Inoculated**	**Direct-contact**	**Aerosol-contact**	**Inoculated**	**Direct-contact**	**Aerosol-contact**
2	6/10	2/10	0/10	5/10	0/10	0/10	5/10	2/10	0/10	0/10	0/10	0/10	3/10	0/10	0/10
4	10/10	10/10	6/10	10/10	5/10	0/10	10/10	10/10	3/10	10/10	2/10	0/10	10/10	6/10	2/10
6	10/10	10/10	8/10	10/10	9/10	0/10	10/10	10/10	8/10	10/10	8/10	0/10	10/10	9/10	5/10
8	9/10	9/10	10/10	6/10	6/10	0/10	8/10	7/10	10/10	8/10	4/10	0/10	10/10	10/10	7/10
10	9/10	7/10	9/10	4/10	3/10	0/10	8/10	5/10	7/10	4/10	2/10	0/10	8/10	7/10	6/10
12	7/10	6/10	7/10	2/10	0/10	0/10	4/10	2/10	3/10	1/10	0/10	0/10	6/10	5/10	4/10
14	5/10	3/10	6/10	0/10	0/10	0/10	0/10	0/10	0/10	0/10	0/10	0/10	3/10	2/10	0/10

*In every independent experiment, ten SPF chickens (inoculated) were infected intranasally 10^7^ EID_50_ of recombinant virus, then another ten animals (direct contact) were introduced into the same isolator 24 h later, while ten animals (aerosol contact) were placed separately in another isolator B. Oropharyngeal and cloacal swab samples of the chickens were collected at 2 day intervals and inoculated in SPF embryonated chicken eggs for observation virus shedding. The result expressed as infected number/total number of chickens. Repeat transmission experiments of r01/NASS and r01/NA381 which could not transmit by aerosols were performed. The results indicate that there was no virus shedding of all aerosol contact animals.

### Mutations D368E, S370L, E313K and G381D in NA of SD01 virus abolished airborne transmission in chickens

The NA protein of influenza A virus is not only required for virion release and spread but also impacts on virion infectivity and membrane fusion [24]. Although studies indicated that amino acid deletion in the stalk region contributes to the high virulence and pathogenicity of H5N1 isolates, in our study, there was no difference between airborne transmissible viruses SD01 and F98, and non-transmissible SS94 which all owned 3-amino acid (aa: 61–63, N2 number) deletion in their NA stalk region. So we investigated whether amino acid changes in the head of the NA protein (aa: 78–469) affected the replication and transmission of mutant viruses in chickens. Multiple sequence alignment of the NA proteins of H9N2 AIV isolated in China and submitted to NCBI from 1994 to 2013 revealed that SS94 and A/Chicken/Guangdong/1997 had acidic amino acid K at position 313, whilst almost all other strains had acidic E313 or D313 [35]. SS94 and three Shandong H9N2 AIV had an acidic D381 residue, while all other viruses had neutral G381 or N381 residues (see Additional file 1). The 3D-structure of neuraminidase was generated using PyMOL software (DeLano Scientific LLC, San Carlos, CA, USA). The G381D mutation was in a random coil region, and the conversion from a non-polar to polar amino acid may result in changes to protein hydrophilicity. When a G381D mutation occurred simultaneously with an E313K mutation in the β-fold region, the protein structure was changed significantly, with the distance between the two reduced amino acids (Figure 1B).

To evaluate whether these amino acid mutations were related to H9N2 virus transmission in chickens, we tested two mutants, r01/NAHB (D368E and S370L) which derives from rSD01 by the two substitutions D368E + S370L in the hemadsorption site, and r01/NA381 (D368E, S370L, E313K and G381D) which derives from r01/NAHB by two further substitutions: E313K + G381D (Figure 1A). r01/NAHB virus shedding was detected in inoculated and direct contact chickens from 2 to 12 dpi, and in aerosol contact chickens, although fewer virus particles were shed compared with rSD01. Virus shedding of r01/NA381 was also detected in inoculated and direct contact chickens, but the number of infected chickens was fewer than those infected by r01/NAHB, and no virus shedding was detected in aerosol contact chickens (Table 2). Seroconversion was observed for all infected chickens (Figures 2C and D), with antibody titers increasing from 7 to 21 dpi. The antibody titers for direct contact or aerosol contact chickens were appreciably lower than for inoculated animals on the same day (Figures 2C and D).

Air samples were collected by an AGI-30 liquid sampler every other day from 2 dpi, and virus titrated in eggs (Figure 3). Virus concentrations were expressed as EID_50_/L air. Viral aerosols were detected for rSD01 and r01/NAHB at concentrations of 265–13330 EID_50_/L air. Generally, airborne H9N2 AIV were first detected at 4 or 6 dpi, peaking at 8 dpi and declining at 10 dpi, with no viral aerosols detected at 12 dpi. Airborne virus was not detected for r01/NASS and r01/NA381, which could be associated with the reduced number of virus aerosols produced, and to assay sensitivity.

Recombinant virus r01/NAHB (which derives from rSD01 by the two substitutions D368E + S370L in the HB site) was detected in the air and was aerially transmitted, which demonstrates that the HB site has a modest impact on aerial transmission. Recombinant virus r01/NA381 (which derives from r01/NAHB by two further substitutions: E313K + G381D) was not detected in the air and was not aerially transmitted, which demonstrates the importance of the amino-acid pair 313–381 in aerial transmission. The NA of this virus is close to that of SS94.

### Replication of recombinant viruses in the respiratory tract of SPF chickens

Clarified homogenates of tracheas and lungs collected from three chickens at 5 dpi were used for virus titration in SPF embryonated chicken eggs. There were no obvious differences in viral titers between the lungs and tracheas of chickens infected with the same virus (Table 3). However, virus titers for r01/NASS and r01/NA381-infected chickens were lower than for those infected with rSD01 (Table 3). According to the study before, these two viruses also had similar characteristics in transmissibility: the reduced virus shedding of infected chickens and no shedding in aerosol contact chickens, and not being detected in the air. All strains were non-lethal to chickens and did not induce serious clinical symptoms, other than slight inappetence and inactivity, with clinical symptoms generally resolved within 10 days. Seroconversion was observed for all viruses, and HI titers were between 64 and 128 (Table 3).

**Table 3:** **Pathogenicity of H9N2 AIV to SPF chickens** ^a^

**Virus**	**Lethality**	**Virus titers in trachea (log**_10_ **EID**_50_ **/gram ± SD)**	**Virus titers in lung (log**_10_ **EID**_50_ **/gram ± SD)**	**Seroconversion (HI titers)**
rSD01	None	5.83 ± 0.29	5.33 ± 0.34	64, 128
r01/NASS	None	3.72 ± 0.35*	3.61 ± 0.26*	64, 64
r01/NAHB	None	5.67 ± 0.34	5.21 ± 0.18	128, 64
r01/NA381	None	4.17 ± 0.29*	3.56 ± 0.10*	128, 64

^a^SPF chickens (*n* = 5) were inoculated intranasally 10^7^ EID_50_ of each virus. Clarified homogenates of tracheas and lungs from three animals collected on 5 dpi were titrated for virus infectivity in SPF embryonated chicken eggs from initial dilutions of 1:10. Virus titers were expressed as mean log_10_EID_50_/g wet tissues ± standard deviation (SD). Two chickens were observed for signs of lethality, and seroconversion was confirmed by hemagglutination inhibition (HI) assay on 14 dpi. *P < 0.05 compared with rSD01.

### Substitutions or mutations in the NA gene affected virus NA activity

Replacement or mutations of the NA gene of SD01 were shown to decrease virus airborne transmission efficiency. In the current study, we determined the kinetic parameters, *K*_M_ and *V*max, of viral NA using the fluorogenic substrate 4-MUNANA. Differences in *K*_M_ values for NA of recombinant viruses were not considered to be statistically significant (*P* > 0.05), suggesting NA enzymatic activity was consistent across all viruses. Enzyme activity was significantly higher (*V*max, *P* < 0.05) for NA of rSD01 compared with NA of r01/NASS, r01/NAHB and r01/NA381 (Table 4). Virus elution times were used to evaluate the effect of NA substitutions or mutations on virus release from CRBC. rSD01 virus was first to be eluted, followed by r01/NAHB, with both r01/NASS and r01/NA381 viruses eluted much later (3.5 and 4 h, respectively). These results indicate that NA gene substitution and mutations at 366–373HB, 313 and 381 played an important role in reducing virus NA activity. What is more, the results also indicate that substitutions at residues 313–381 have more impact than those at the HB site.

**Table 4:** **Neuraminidase activities of H9N2 AIV**

**virus**	***K***_M_ **(μM)** ^a^	***V***_max_ **(fluorescence a.u/s)** ^a^	**Relative** ***V***_max_^b^	**Elution time(h)**
rSD01	54.16 ± 3.12	192.73 ± 6.54	1.00	1.5
r01/NASS	49.167 ± 3.34	118.83 ± 1.43*	0.62	3.5
r01/NAHB	52.43 ± 6.68	123.83 ± 3.40*	0.64	2
r01/NA381	53.84 ± 2.53	92.25 ± 1.69*	0.48	4

^a^The enzyme kinetics data was fit to the Michaelis-Menten equation by nonlinear regression to determine the Michaelis constant (*K*_M_) and maximum velocity (*V*max) of substrate conversion. Results are given as the mean ± standard deviation from three duplicate samples. ^b^Relative *V*max: homologous of recombinant virus to SD01. *P < 0.05 compared with SD01.

### Mutations E368D, L370S, K313E and D381G in NA of r01/NASS confer virus airborne transmissibility in chickens

r01/NASS-381 virus with mutations E368D, L370S, K313E and D381G in NA was generated on the backbone of the r01/NASS virus which had lost airborne transmissibility in chickens. We compared the transmissibility of r01/NASS-381 virus with r01/NASS virus in chickens. As shown in Table 2, virus shedding was detected in both r01/NASS-381 and r01/NASS inoculated and direct contact chickens, with a greater number of chickens infected by the former from 4 to 14 dpi. Two aerosol contact chickens were infected with r01/NASS-381 virus at 4 dpi; increasing to seven by 8 dpi. Altogether, fewer chickens were infected with r01/NASS-381 compared with rSD01 and r01/NAHB on the same day. Seroconversion occurred in aerosol infected chickens, with average antibody titers of 4–32 recorded at 14 and 21 dpi (Figure 2E). Air samples were collected from the isolators and viruses titrated in SPF embryonated chicken eggs, as described previously. Airborne r01/NASS-381 virus was detected at 6 dpi, at a concentration of 265 EID_50_/L air, whilst no viral aerosol was detected at 12 dpi (Figure 3). Virus elution times of r01/NASS-381 virus released from CRBC was 2.5 h, which was shorter than that of the r01/NASS virus. These results demonstrate that amino acid mutations E368D, L370S, K313E and D381G in the NA protein of r01/NASS enhanced viral NA activity and viral shedding from chickens, and restored airborne transmissibility in chickens, and the HB site had a minor impact on these characteristics, while the amino-acid pair 313–381 had a major effect.

## Discussion

H9N2 AIV was first isolated from dead chickens in the Guangdong Province, China, in 1994, and in the proceeding decades has spread widely throughout other provinces, impacting heavily on the poultry industry and imparting great economic losses [2,10,36]. A number of studies have demonstrated the capability of AIV to cause aerosol infections by the respiratory route, moreover, AIV infections occurred between animals through the airborne route in many experimental models [37,38]. The high prevalence of H9N2 AIV in poultry is also associated with airborne transmission. Aerosol transmission of H9N2 AIV was observed in SPF chicken flocks, and recovered airborne virus particles [22]. Shi et al. demonstrated that the F98 virus could be transmitted through chicken flocks by aerosols, whilst the SS94 virus could not. The authors discovered that the mechanism of airborne transmission was associated with the NA gene [23]. To elucidate the mechanisms underlying airborne transmission mechanisms of H9N2 AIV, reverse genetics technology should be employed to establish the relationship between NA amino acids and transmissibility of AIV.

Influenza virus NA is an important viral surface glycoprotein, possessing exoglycosidase activity, catalyzing the hydrolysis of α-glycosidic bonds between sialic acid residues and adjacent oligosaccharides. NA plays an important role in the virus life cycle, including invading and infecting cells, budding and release, and preventing self-aggregation [24,39-41]. Richard et al. reported that D198N, E119D and I222L mutations in NA of H3N2 influenza virus could alter the biological activity of NA [42]. While a substitution of the NA gene from the 2009 H1N1 influenza virus, the recombinant virus (other genes from H1N1 seasonal influenza virus) failed to increase virus replication in vivo. However, the transmission of droplet infection of the recombinant virus was significantly enhanced among mammals [33]. In a previous study, we demonstrated that the SD01 virus could transmit in chickens by airborne route using the isolator apparatus [22]. To investigate the relationship between the NA gene and virus airborne transmission, a reverse genetics system was employed to rescue recombinant H9N2 AIV. We found that the recombinant virus lost parental airborne transmissibility in chickens when the NA gene of the SD01 virus was substituted with that of the SS94 virus. These results were consistent with the work of Shi et al. [23]. We used DNASTAR software to establish that amino acid residues 313, 331, 366–373 and 381 in the active region of NA differed between airborne transmissible viruses SD01 and F98, and non-transmissible SS94. The aim of our present study was to investigate the role of these amino-acids in conferring the airborne transmission ability.

In the present study, SPF chickens inoculated with r01/NAHB virus, continued to infect aerosol contact chickens, and viral aerosols were recovered from the air of isolators. These results indicate that r01/NAHB virus could still transmit in chickens via the airborne route. The replication capacity of r01/NAHB virus within the trachea and lung tissue of SPF chickens did not differ significantly from rSD01 virus. However, NA activity was lower for the r01/NAHB virus, and time of virus release from CRBC was greater. Therefore, NA activity of H9N2 AIV was affected by amino acid mutations at the HB site, although these did not severely impact the transmission and replication of the virus.

Other studies have also shown that amino acid mutations on NA affect its activity. It was found that the NA activity of A/Moscow/10/99 (H3N2) influenza virus with D198N and E119D mutations in NA was considerably lower than for the parental virus following stable passage in MDCK cells [42]. Some NA amino acids have been shown to play a significant role in airborne transmission of influenza virus. Herlocher et al. reported that substitution of R292K in the NA of A/Sydney/5/97 weakened virus infectivity and abolished parental airborne transmissibility in ferrets. They also demonstrated that substitution H274Y could reduce the transmission ability of H1N1 influenza virus in mammals [43,44]. In the present study, SPF chickens inoculated with a recombinant virus r01/NA381 (containing mutations D368E, S370L, E313K and G381D in SD01 virus NA) failed to infect chickens in the aerosol contact group, and airborne virus was not detected in the isolators. Collectively, these results suggest that r01/NA381 had lost its capacity for airborne infection. The protracted elution of r01/NA381 from CRBC, coupled with a reduction in NA activity, and decreased replication capacity in the respiratory tract of chickens may be important factors in the loss of airborne infection capability of this virus.

r01/NASS (rSD01 containing the SS94 NA gene) and SS94 virus transmission does not proceed via the airborne route in chickens. The introduction of four mutations, E368D, L370S, K313E and D381G, in NA of r01/NASS conferred transmissibility of the recombinant virus by aerosols in chickens, especially, the amino-acid pair 313–381 had a major effect on airborne transmissibility. These amino acid mutations in NA of the SS94 virus, illustrate the potential for conferral of airborne virus transmissibility in poultry, which could lead to rapidly spreading epidemics, and a considerable threat to poultry and the industry it supports.

In recent years, several subtypes of AIV, including H3, H5, H6, H7 and H9, have been circulating and evolving in China [45-47]. In such an environment, AIV can easily recombine with other subtypes to generate new viruses. Recombination of airborne transmissible H9N2 virus with highly pathogenic AIV such as H5 and H7 subtypes, could produce recombinant viruses which are both highly pathogenic and airborne transmissible. Therefore, further research should be directed towards airborne transmission of the H9N2 virus.

In conclusion, we demonstrate that H9N2 AIV NA was important for airborne transmission of virus in poultry. Specifically, residues E368, L370, K313 and D381 of NA contribute to NA activity and replication capacity in the respiratory tract of poultry. More importantly, these amino acids, especially K313 and D381, play a role in preventing virus airborne transmission. During the submission process, Zhong et al. reported the critical roles of HA and PA in the airborne transmission of a couple of H9N2 viruses [48]. These findings can be applied to guide the transmission ability assessment of H9N2 AIV and forecast disease outbreaks in the coming years.

## Additional file

Additional file 1:
**Multiple sequence alignment of the NA proteins of H9N2 AIV.** The viruses used for alignment were isolated in China and submitted to NCBI from 1994 to 2013.
