# Supplementary figures and images for: Amino acid substitutions in the neuraminidase protein of an H9N2 avian influenza virus affect its airborne transmission in chickens
Source: Vet Res. 2015 Apr 18;46(1):44. doi: 10.1186/s13567-014-0142-3 (PMC4404070; doi:10.1186/s13567-014-0142-3)

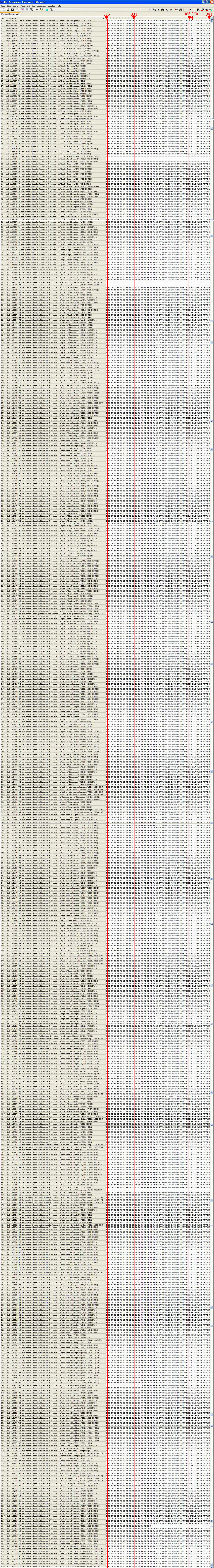

Supplement: Additional file 1: — Multiple sequence alignment of the NA proteins of H9N2 AIV. The viruses used for alignment were isolated in China and submitted to NCBI from 1994 to 2013. [file 13567_2014_142_MOESM1_ESM.jpeg]
